# *Haplopelma hainanum* venom induces inflammatory skin lesions

**DOI:** 10.7717/peerj.8264

**Published:** 2020-01-10

**Authors:** Zhili Deng, Yaling Wang, Wei Shi, Lei Zhou, San Xu, Ji Li, Yiya Zhang

**Affiliations:** 1 Department of Dermatology, Xiangya Hospital, Central South University, Changsha, China; 2 Key Laboratory of Organ Injury, Aging and Regenerative Medicine of Hunan Province, Central South University, Changsha, China; 3 Center for Molecular Medicine, Xiangya Hospital, Central South University, Changsha, China; 4 National Clinical Research Center for Geriatric Disorders, Xiangya Hospital, Central South University, Changsha, China

**Keywords:** *H. hainanum* venom, Envenomation, Inflammation, Revascularization

## Abstract

The *Haplopelma hainanum* is a species of theraphosid spider from China. Its large size and charming appearance make this species a popular pet. According to a previous study, theraphosid spider bites can induce pain, erythema, and edema in humans and can present more severely in domestic animals. The pathological consequences of envenomation by *H. hainanum* remain unclear. In this study, we investigated the effects and mechanisms of *H. hainanum* envenomation in mice. We showed that the venom induced slight swelling, intense inflammatory response, and increased the microvascular density in mice skin. Moreover, we found that 50 µg/ml of the spider’s venom induced IL-1β expression in both HaCaT cells and fibroblast cells, but repressed CXCL10 expression in fibroblasts. The venom significantly induced cell senescence and repressed cell proliferation and migration in both HaCaT cells and fibroblast cells. Finally, we examined the expression of Nav channel in HaCaT and fibroblast cells and found that *H. hainanum* venom effectively inhibited Na^+^ currents in HaCaT cells. Our study calls for further investigation of the pathological consequences and potential mechanisms of *H. hainanum* envenomation. This information might assist in the development of suitable therapy.

## Introduction

Spiders are one of the oldest and most abundant venomous animals, with a fossil history spanning more than 300 million years and over 40,000 species (Deng et al., 2016). Every year, approximately 10,000 spider bites are reported in Brazil and nearly 3,000 bites in America (Braitberg & Segal, 2009).

The venom of most spiders causes only minor discomfort including edema, hemorrhage, and sometimes subsequent ulceration (Dunbar et al., 2018; Isbister & Fan, 2011). Though relatively rare, spider envenomation also can cause severe reactions such as systemic loxoscelism, which can progress to acute renal failure and even death (Manzoni-de Almeida et al., 2018; Okamoto et al., 2017). Most studies on spider envenomation focus on one of the most venomous spiders, the *Loxosceles*. Studies have shown that histopathologic alterations induced by *Loxosceles* envenomation include edema, vasodilatation, and hemorrhage in dermis–epidermis dissociation (Van den Berg et al., 2007). Also, the complement system plays an important role in envenomation-induced inflammation (Patel et al., 1994; Ribeiro et al., 2015; Tambourgi et al., 2005). An increasing number of studies have revealed the important role of fibroblasts and keratinocytes in spider venom-induced pathological alterations in the skin. *Loxosceles* envenomation partly induces dermonecrosis by upregulating proinflammatory cytokine expression in fibroblasts (Dragulev et al., 2007; Rojas et al., 2017). Another study showed that keratinocyte-secreted matrix metalloproteinase contributed to the induction of dermonecrosis by both *Loxoscles laeta* and *Loxosceles intermedia* venom (Correa et al., 2016). Moreover, *Loxosceles* venom triggers cell death by apoptosis in human skin fibroblasts (Dantas et al., 2014) and keratinocytes, contributing to the pathogenesis of cutaneous loxoscelism (Paixao-Cavalcante & Van den Berg, 2006).

Theraphosid spiders, also called bird spiders, are increasingly being kept as pets due to their size and beautiful coloring (Fuchs et al., 2014). Although theraphosid spiders are considered harmless, their venom has been proven to cause localized pain, erythema, and edema in humans, with more severe symptoms in canines, including death (Isbister et al., 2003; Rocha et al., 2016). *Haplopelma hainanum* is a venomous species of theraphosid spider from the Hainan province in southern China (Xiao & Liang, 2003). Previous studies have focused on the peptides in *H. hainanum* venom that directly regulate the activation of ion channels, producing analgesic effects (Zhang et al., 2015). The histopathologic alterations caused by *H. hainanum* envenomation, however, are virtually unknown.

In this study, we examined the pathological alterations induced by *H. hainanum* venom in mice, and discovered the mechanism that potentially contributes to lesion development in HaCaT and fibroblast cells. We developed an understanding of the action of the molecular mechanisms of *H. hainanum* venom, which may assist in the development of various treatments aimed at ameliorating the symptoms of spider envenomation.

## Materials & Methods

### Animals

Twenty female C57BL/6 mice (8 weeks old) were used in this study. All mice received food and water prior to the experiment with a 12 h/12 h day/night cycle. All animal experiments were approved by the Animal Care and Use Committee of the Xiangya Hospital of Central South University (201703211).

### Spider venom and treatment

The venom was collected from adult female *H. hainanum* using an electro-pulse stimulator as described previously (Hu et al., 2014; Yan et al., 2018). Expelled venom was collected from the fang tips with a tube, pooled, and freeze-dried. The freeze-dried crude venom was stored at −20 °C prior to analysis. *H. hainanum* venom (0, 1, 3, 10 and 30 µg/site) was injected into the ear in a fixed volume of 25 µl in PBS. 24 h after the intradermal (i.d.) injection of venom, the skin was collected and stored at −80 °C.

### Cell culture and treatment

Human keratinocyte HaCaT cells were purchased from the Cell Bank of Chinese Academy of Sciences (Shanghai, China). The primary human skin fibroblast cells were cultured by digesting human skin with type II collagenase (Sigma, Aldrich, St. Louis, MO, USA) to isolate human keratinocytes as described previously (Xie et al., 2013). Fibroblast cells were cultured in Dulbecco’s modified Eagle’s medium (DMEM, Hyclone, Logan, USA) with 10% fetal bovine serum (Hyclone, Logan, UT, USA) as previously described (Li et al., 2010). The human keratinocyte HaCaT cells were cultured in free-calcium basal medium (DMEM; Gibco, USA) with 10% fetal bovine serum (Hyclone, Logan, USA) as previously described (Li et al., 2019) in an incubator at 37 °C, 5% CO_2_.

### Histologic analysis

Mice ears were sectioned at 4 µm thickness and then stained with hematoxylin and eosin (H&E) (four ears per group). The histological alterations were detected by microscopy (OLYMPUS, Japan).

### Immunofluorescence

The ears were sectioned at 8 µm thickness and incubated with anti-CD4, anti-CD31, and anti-MHCII antibodies (4 ears per group), and then stained with anti-goat IgG antibodies (Alexa Fluor 488) as previously described (Su et al., 2018). All antibodies were purchased from ebioscience (San Diego, USA).

### Real-time PCR analysis

A TRIzol reagent (Invitrogen Life Technologies) was used to derive the total RNA from HaCaT and fibroblast cells. Two µg of RNA was reverse transcribed to cDNA. We then performed qPCR to obtain mRNA expression as previously described (Li et al., 2019). The primers of IL-1β, CXCL10, TNF-α, IL-6, IL-17, and CCL2 are listed in Table 1. The real-time PCR analysis was repeated in three independent experiments.

**Table 1:** Primers for qRT-PCR.

Primier	Forward (5′ to 3′)	Reverse (5′ to 3′)
IL-1β	AGCTACGAATCTCCGACCAC	CGTTATCCCATGTGTCGAAGAA
CXCL10	GTGGCATTCAAGGAGTACCTC	TGATGGCCTTCGATTCTGGATT
TNFα	CCTCTCTCTAATCAGCCCTCTG	GAGGACCTGGGAGTAGATGAG
IL-6	CCTGAACCTTCCAAAGATGGC	TTCACCAGGCAAGTCTCCTCA
IL-17	TCCCACGAAATCCAGGATGC	GGATGTTCAGGTTGACCATCAC
CCL2	CAGCCAGATGCAATCAATGCC	TGGAATCCTGAACCCACTTCT

### Cell migration

The cell migration ability was detected using a scratch-wound assay. Cells were seeded and cultured in a 6-well plate. When cells reached ∼80% confluence, a 100 µl tip was used for scratching. The cells were treated with 0, 5, 10, 20, 50, and 100 µg/ml venom in 5% FBS DMEM for 12 h. The cell wound conditions were then photographed using the Zeiss Axio Scope A1 microscope (Zeiss, Oberkochen, Germany). This was repeated 4 times.

### Cell senescence

Senescence was detected using SA-β-gal staining as previously described (Xie et al., 2017). When cells reached 70% confluence, SA-β-gal solution was used for staining. β-Gal-positive cells were detected using microscopy (OLYMPUS, Japan). This was repeated in three independent experiments.

### Cell proliferation

The cell proliferation ability was assessed using an MTT assay. 1 × 10^4^ cells were seeded in 96-well plates and cultured for 24 h, 48 h, and 72 h. MTT (Sigma-Aldrich) and dimethyl sulfoxide (DMSO) (Sigma-Aldrich) were added to the 96-well plates and the absorbance was measured at 490 nm. This was repeated in 5 replicates per experiment and in 3 independent experiments.

### Patch clamp

The solidum currents in HaCaT and fibroblast cells were detected using the whole-cell patch-clamp technique (Axon 700B patch-clamp, Irvine, CA, USA) as previously described (Yan et al., 2018). To detect voltage-gated Nav currents, we used an extracellular solution containing (in mM): 145 NaCl, 1.5 CaCl_2_, 2 MgCl_2_, 2.5 KCl, 10 D-glucose and 10 HEPES, (pH 7.4). The pipette solution contained (in mM): 5 NaCl, 135 CsCl, 5 MgATP, 10 D-glucose, 10 HEPES and 10 EGTA (PH 7.2). The current was elicited by -10 mV from a holding potential of −40 mV. This was repeated for 3 different experiments.

### Statistical analysis

GraphPad Prism 6 (La Jolla, CA) was used for statistical analysis. Data were presented as means ± SEM. Statistical comparisons of the two groups were analyzed by the Student’s *t*-test. *P* < 0.05 was considered significant as compared to the control group.

## Results

### Histological assessment and inflammatory cell infiltration of skin damage

**Figure 1:**
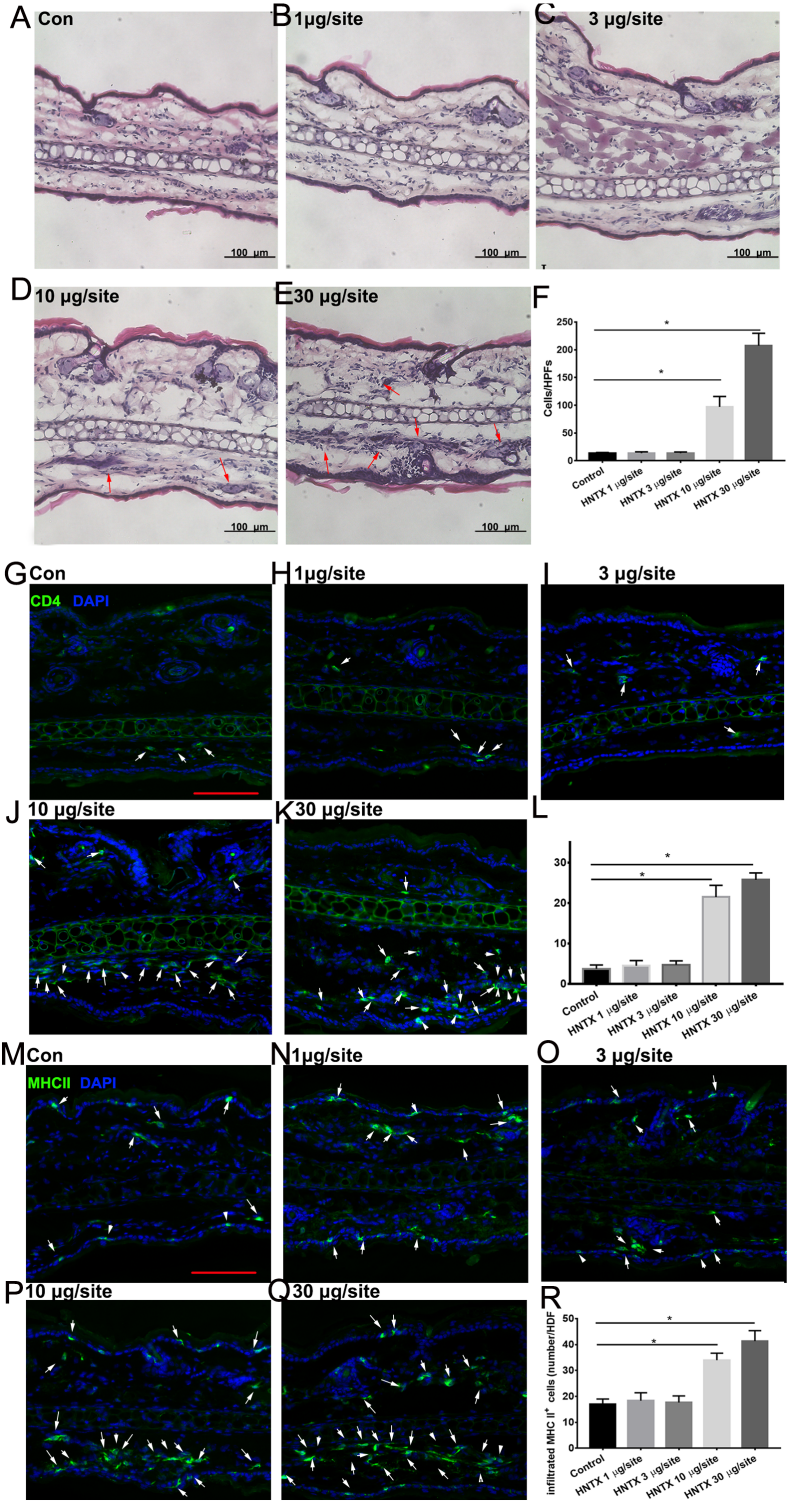
Histological alterations in *H. hainanum.* venom induced skin lesion. (A–F) H&E stain revealed the histological alterations. The arrows are inflammatory cells. (G–L) Immunofluorescence revealed the CD4^+^ T cells infiltration. The arrows are CD4+ cells. Scale bar: 100 µm. (M–R) Immunofluorescence revealed the MHCII^+^ cells infiltration. The arrows are MHCII^+^ cells. Scale bar: 100 µm. Data represent the means SEM.*P < 0.05, compared to control group.

Histopathological analysis of the mice’s ear skin 24 h after i.d. *H. hainanum* venom injection showed histological alterations including slight swelling and an intense leukocyte infiltrate in which neutrophils were the predominant cell type deep in the dermis (Figs. 1A–1F). We also detected the infiltration of CD4^+^ T cells (CD4^+^) and APCs (MHCII^+^) using immunofluorescence staining. As shown in Figs. 1G–1L, the number of CD4^+^ T cells significantly increased in the lesions where *H. hainanum* venom was applied at 10 µg per site and 30 µg per site. Moreover, the number of MHCII^+^ cells also increased at the 10 µg per site and 30 µg per site where venom was applied (Figs. 1M–1R). Together, these results indicate that *H. hainanum* venom induces inflammatory cell infiltration in mice.

### Alterations in the microvascular density

**Figure 2:**
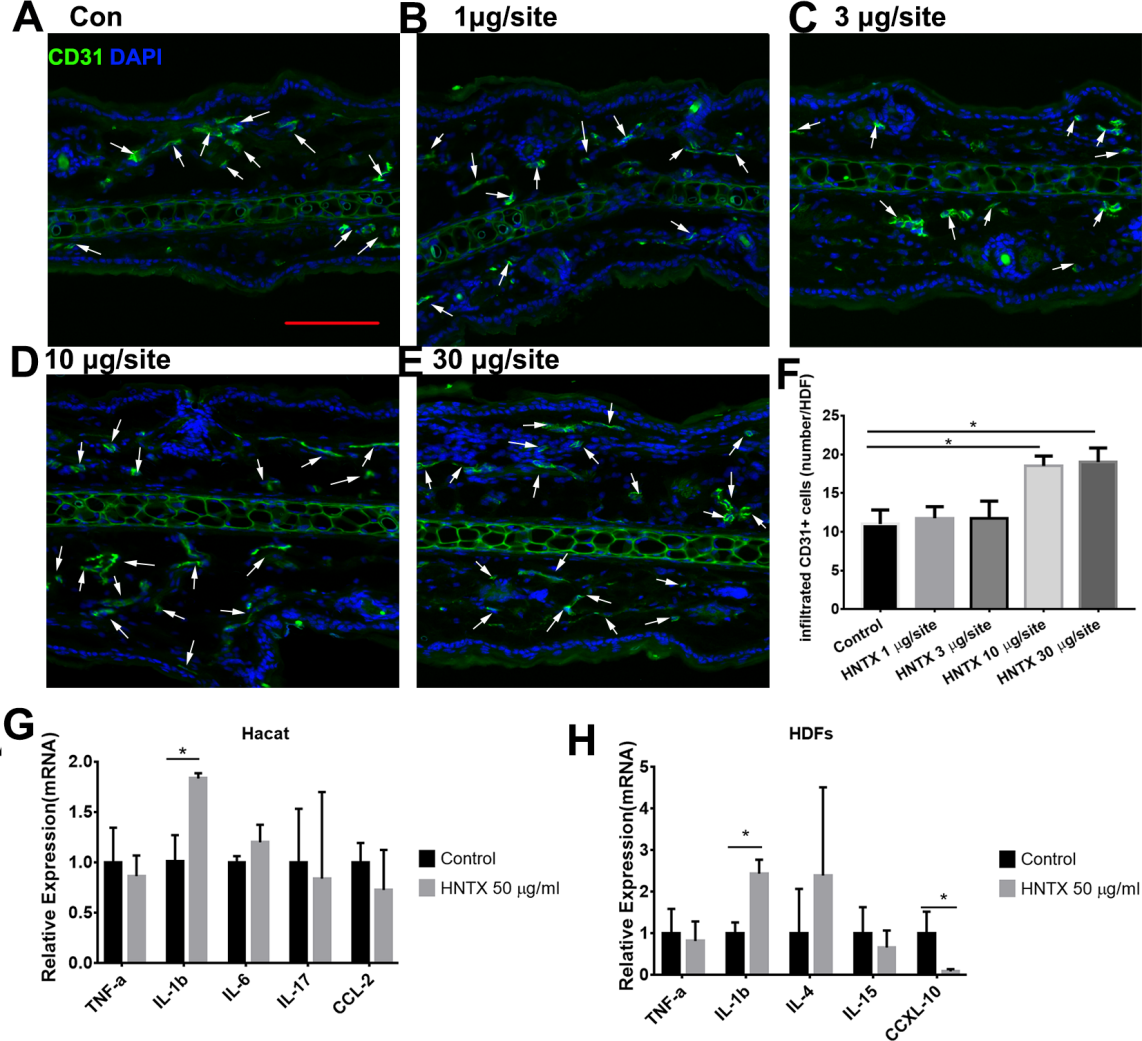
The effects of *H. hainanum* venom on the alterations in the microvascular density in mice and inflammatory cytokines in keratinocytes and fibroblasts. (A–F) CD31^+^ cells were detected using immunofluorescence. Scale bar: 100 µm. The arrows are CD31^+^ cells. Data represent the means ± SEM. *P < 0.05, compared to control group. (G) qPCR revealed the expression of inflammatory cytokines in keratinocytes. (H) qPCR revealed the expression of inflammatory cytokines in fibroblasts. Data represent the means ± SEM. *P < 0.05, compared to control group.

Previous studies have shown alterations in microvascular density in mice treated with snake venom treated (Jimenez et al., 2008) Here, we demonstrated the role of *H. hainanum* venom on microvascular density by detecting CD31^+^ cell using immunofluorescence. As shown in Figs. 2A–2F, at 10 µg per site and 30 µg per site venom evidently increased the number of CD31^+^ cells. These results indicate that *H. hainanum* venom induces revascularization.

### Venom induces the production of inflammatory cytokines

Keratinocytes, when provoked by environmental stimuli, can produce inflammatory cytokines and chemokine (Hermann et al., 2017; Lowes et al., 2013; Zhang et al., 2016). Moreover, dermal fibroblasts produce cytokines and antimicrobial peptides to defend against pathogens (Hesse-Macabata et al., 2019). In this study, we assessed the effects of *H. hainanum* venom on inflammatory cytokine expression in HaCaT cells and fibroblast cells. As shown in Fig. 2G, 50 µg/ml venom evidently induced IL-1β expression in HaCaT cells. Moreover, 50 µg/ml venom induced IL-1β expression and repressed CXCL10 expression in fibroblasts (Fig. 2H). TNF-*α*, IL-6, IL-17, and CCL2, however, were not affected by *H. hainanum* venom. Together, these results indicate that venom partly induces inflammatory infiltration by upregulating IL-1β expression in both HaCaT cells and fibroblast cells.

### The effects of venom on the proliferation, migration, and senescence of keratinocytes

**Figure 3:**
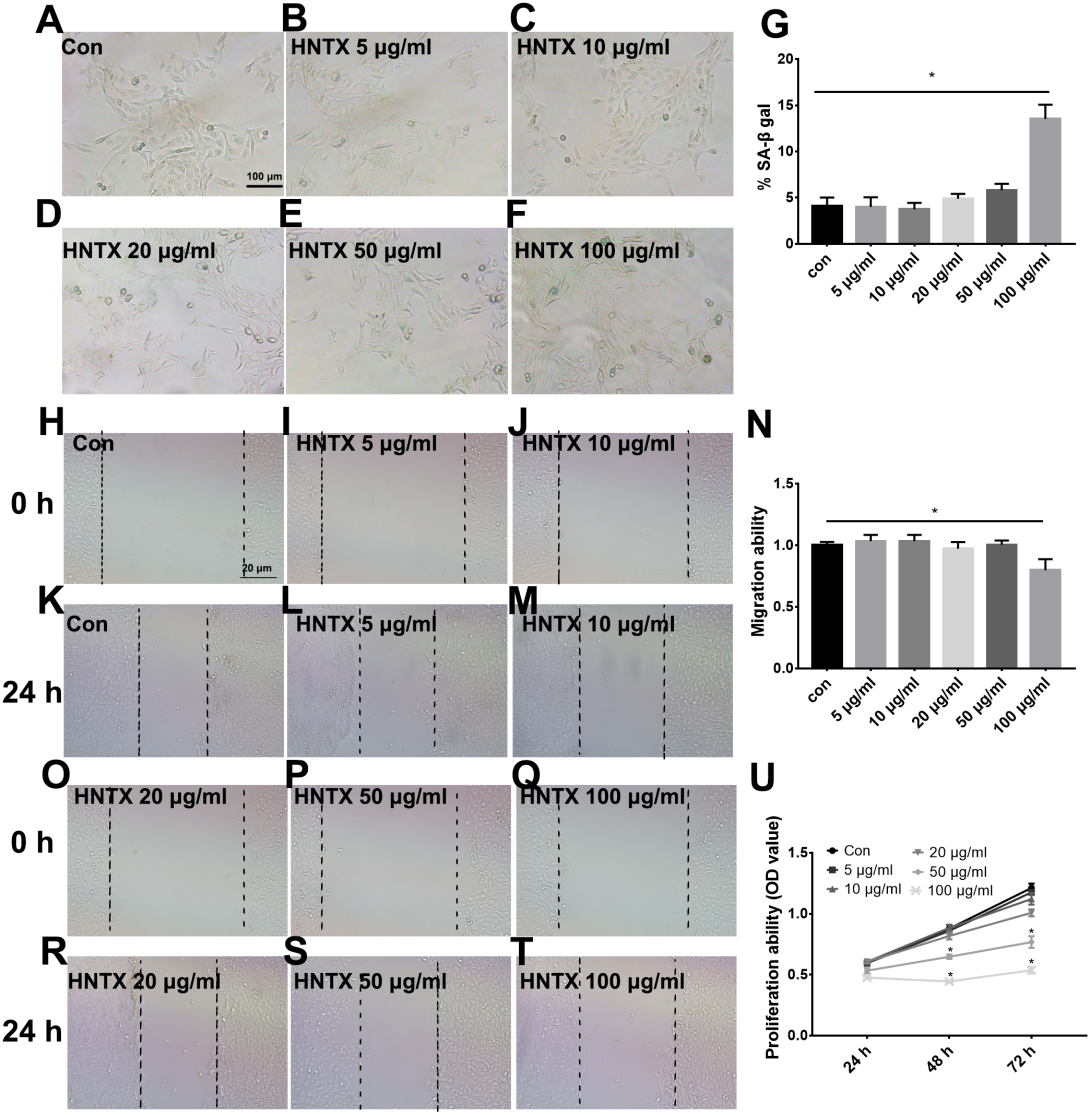
The *H. hainanum* venom affects proliferation, migration and senescence of keratinocytes. Cells were treated with 0, 5, 10, 20, 50 and 100 µg/ml venom. (A–G) SA-β-gal staining was used to determinate cell senescence of keratinocytes. (H–T) wound healing was used to determinate cell migration of keratinocytes. U, MTT was used to determinate cell proliferation of keratinocytes. Data was represented as the means ± SEM. *P < 0.05, compared to control group.

Keratinocytes are the major component of the epidermis, which plays a central role in maintaining the barrier function of the skin. In this study, we treated HaCaT cells with 0, 5, 10, 20, 50, and 100 µg/ml *H. hainanum* venom and then determined cell proliferation, migration, and senescence using MTT, wound healing, and SA-β-gal staining, respectively. As shown in Figs. 3A–3G and 3H–3T, 5, 10, 20, and 50 µg/ml *H. hainanum* venom did not significantly affect cell senescence or repressed cell migration ability. 100 µg/ml *H. hainanum* venom evidently induced cell senescence and repressed the migration ability of HaCaT cells. As shown in Fig. 3U, 50 and 100 µg/ml *H. hainanum* venom significantly repressed the cell proliferation ability of HaCaT cells. Five, 10, and 20 µg/ml *H. hainanum* venom did not seem to impact HaCaT cell proliferation. In conclusion, the data demonstrate that a high enough concentration of *H. hainanum* venom directly affects the proliferation, migration, and senescence of keratinocytes.

### The effects of venom on the proliferation, migration, and senescence of fibroblasts

**Figure 4:**
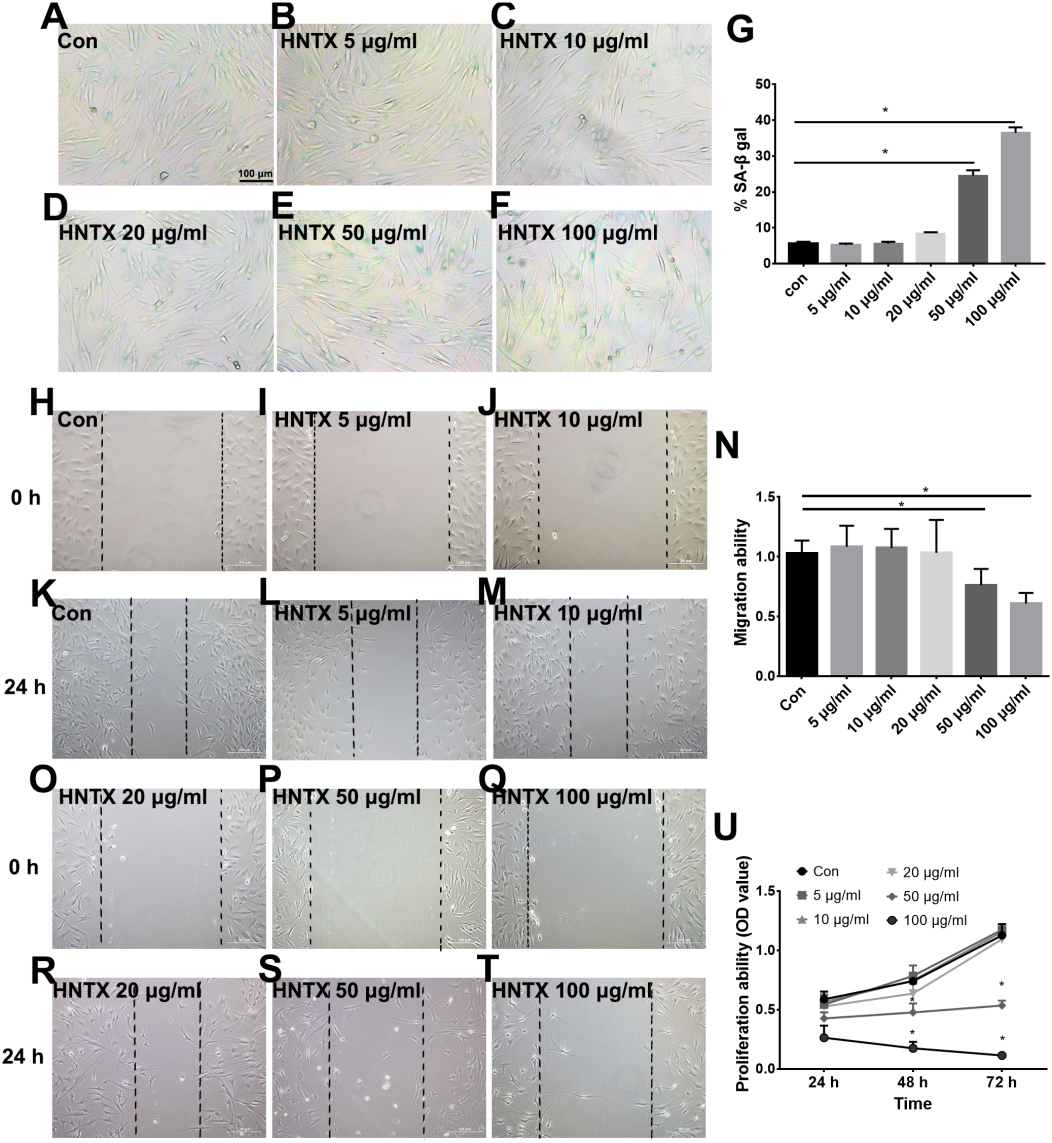
The *H. hainanum.* venom affect proliferation, migration and senescence of fibroblasts. (A–G) SA-β-gal staining was used to determinate cell senescence of fibroblasts. (H–T) wound healing was used to determinate cell migration of fibroblasts. (U) MTT was used to determinate cell proliferation of fibroblasts. Data represent the means ± SEM. *P < 0.05, compared to control group.

Next, we explored the potential role of *H. hainanum* venom on fibroblast cell proliferation, migration, and senescence using MTT, wound healing, and SA-β-gal staining, respectively. As shown in Figs. 4A–4U, 50 µg/ml and 100 µg/ml *H. hainanum* venom evidently induced cell senescence and repressed the cell migration and proliferation ability of fibroblast cells. Five, 10, and 20 µg/ml *H. hainanum* venom did not significantly affect cell proliferation, migration, or senescence (Fig. 4). In conclusion, these data demonstrate that a high enough concentration of *H. hainanum* venom directly affects the proliferation, migration, and senescence of fibroblasts.

### Venom inhibits the currents of voltage-gated sodium channels in HaCaT and fibroblast cells

**Figure 5:**
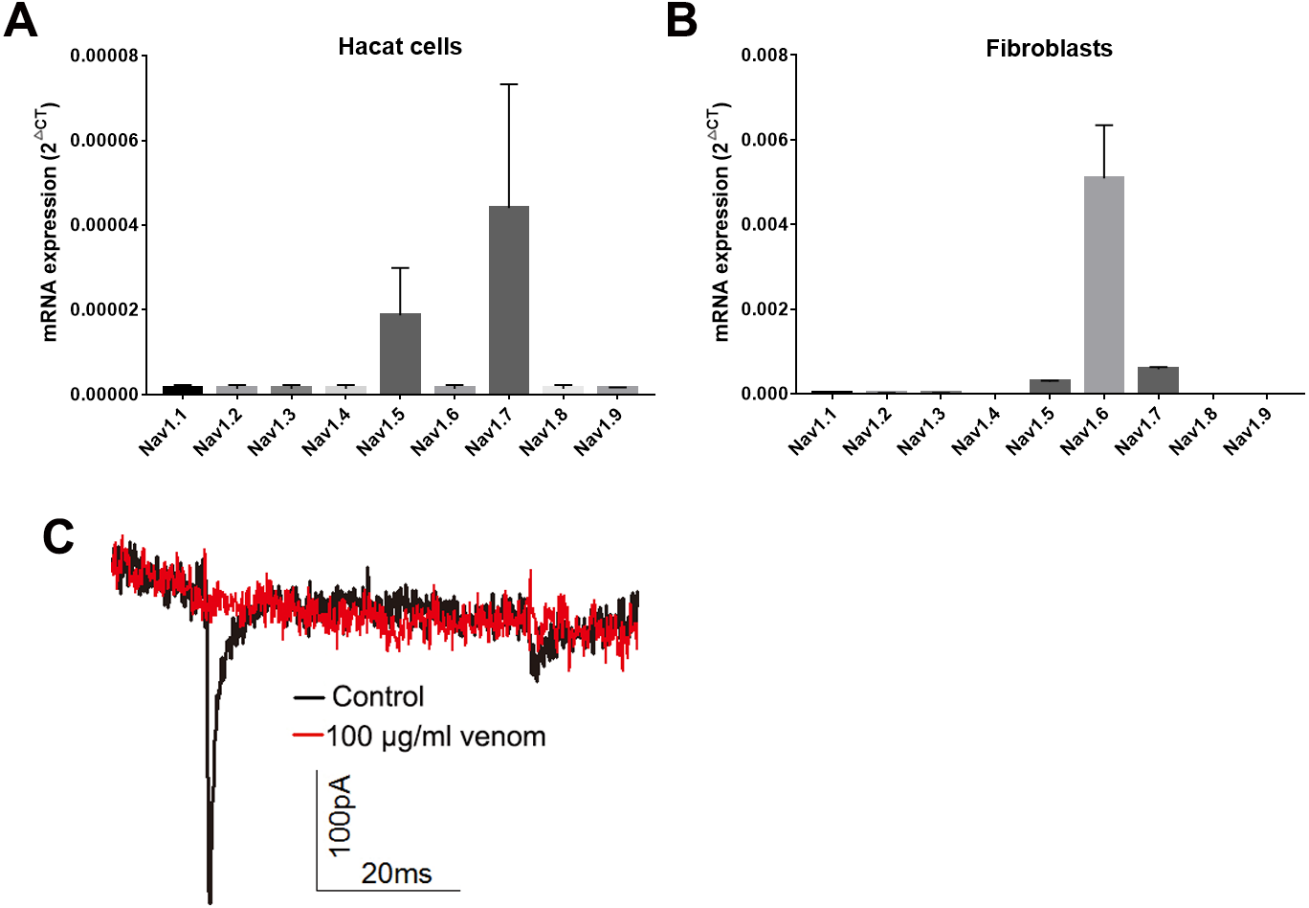
Venom inhibits the currents of voltage gated sodium channels in Hacat cells. (A) The expression levels of sodium channels in Hacat. (B) The expression levels of sodium channels in fibroblasts. (C) The sodium currents on Hacat using patch clamp. Data represent the means ± SEM.

Studies have shown that keratinocytes and fibroblasts have sodium channels expression (Zhao et al., 2008). In this study, we determined the expression levels of sodium channels in HaCaT and fibroblasts cells. In HaCaT cells, the Nav1.5 and Nav1.7 expression levels were higher than those of other sodium channels (Fig. 5A). In fibroblast cells, the Nav1.5, Nav1.6 and Nav1.7 expression levels were higher than those of other sodium channels (Fig. 5B). We next detected the sodium currents of HaCaT and fibroblast cells using a patch clamp. As shown in Fig. 5C, voltage-gated sodium currents were detected in HaCaT cells, as 100 µg/ml venom evidently repressed the sodium currents. Unfortunately, no sodium currents were detected in fibroblasts.

## Discussion

In humans, envenomation by theraphosid spiders can result in painful skin lesions that include erythema and edema (Rocha et al., 2016). The object of this study was to investigate the potential role of *H. hainanum* venom in the pathology of such skin lesions *in vivo* and *in vitro*. We found that *H. hainanum* venom induces effects that closely mimic the envenomation-induced lesions in humans. The histological alterations include slight swelling, inflammatory cell infiltration, and revascularization. Moreover, *H. hainanum* venom evidently induced the production of proinflammatory cytokines and significantly affected the cell proliferation, migration, and senescence in keratinocytes and fibroblasts.

Spider venom is a complex cocktail of proteins, neurotoxic peptides, and small molecules. It is used to capture prey and defend against predators (Windley et al., 2012). Although the bite of most spiders has little or no effect on mammalian tissue, envenomation by *Loxosceles*, for example, can induce localized pain, erythema, edema and dermonecrosis in skin. Spider envenomation can also induce severe systemic reactions, even death (Manzoni-de Almeida et al., 2018; Okamoto et al., 2017). For example, *Loxosceles* venom can induce edema, vascular anomalies, hemorrhaging, and dermis–epidermis dissociation (Mackinnon & Witkind, 1954). Spider venom can also induce a severe inflammatory response, including an increase of proinflammatory cytokines and chemokines and leukocyte infiltration (Dunbar, Sulpice & Dugon, 2019; Griesbacher et al., 1998; Rojas et al., 2017; Tambourgi et al., 2005). In this study, we examined the envenomation of mice with *H. hainanum* venom. Consistent with the clinical presentation of theraphosid spider bites in human (Rocha et al., 2016), *H. hainanum* venom injection caused histological alterations including slight swelling, an intense leukocyte infiltrate, and increased microvascular density in mice (Figs. 1 and 2). Studies have shown that phospholipases D is an important active constituent of venom in spiders from the *Sicariidae* family (Lopes et al., 2013). It plays a proinflammatory role by activating leukocytes (Manzoni-de Almeida et al., 2018) in the progression of the dermonecrotic lesion (Paixao-Cavalcante et al., 2007). There was no phospholipases D, however, detected in the venom of theraphosid spiders. Moreover, LmTX-I (a basic phospholipase A2) is considered an important component of *Lachesis muta muta* venom that induces microvascular permeability in skin (Ferreira et al., 2009). Kinin-related peptides were reported to play a key role in *Vespula vulgaris* venom, inducing paw edema in rats (Griesbacher et al., 1998). Various neurotoxic peptides in theraphosid spider venom attracts enormous interest, given their potential for pharmacological use in regulating the activation of various ion channels (Zhang et al., 2015). Our previous studies demonstrated that hainantoxin-I, a peptide toxin in *H. hainanum* venom, is an activator of the KCa3.1 channel (Huang et al., 2014). The channel modulates Ca(^2+^) influx and plays a key role in the activity of various immune cells, including mast cells, inflammatory CD4^+^ T, and antigen-specific memory T cells (Chiang et al., 2017; Duffy et al., 2015; Matsui et al., 2018). These results indicate that hainantoxin-I may be involved in the inflammation induced by *H. hainanum* venom.

Keratinocytes are not merely a major component of the skin’s physical barrier. They are also critical for innate immunity, producing a variety of proinflammatory cytokines that play a key role in the activation and recruitment of immune cells in the skin (Kabashima et al., 2019). Fibroblasts, which are the major cells for the production of collagen and the most abundant component of the ECM in the dermis, are also reported to contribute to immune defense by producing proinflammatory cytokines and antimicrobial peptides (Hesse-Macabata et al., 2019). Previous studies have shown that *Loxosceles* venom induced the production of proinflammatory cytokines and chemokines in endothelial cells and fibroblasts (Desai et al., 1999; Gomez et al., 1999; Rojas et al., 2017). Our work has demonstrated that *H. hainanum* venom evidently induced IL-1β expression in both HaCaT cells and fibroblast cells. The expression of TNF-α, IL-6, IL-17, and CCL2, however, was not affected by *H. hainanum* venom. In conclusion, these results indicated that *H. hainanum* venom-induced inflammation was partly caused by the production of cytokines in HaCaT and fibroblast cells. Additionally, *H. hainanum* venom evidently affected biological functions including cell senescence, migration, and proliferation in both HaCaT cells and fibroblasts. Fibroblasts showed more sensitivity to *H. hainanum* venom than HaCaT cells did. It has previously been shown that *Loxosceles* venom induced cell apoptosis of human keratinocytes, which is consistent with our results for *H. hainanum* venom (Paixao-Cavalcante & Van den Berg, 2006). Other studies have shown that that keratinocytes and fibroblasts have sodium channel expression (Zhao et al., 2008). In this study, we showed that Nav1.5 and Nav1.7 were highly expressed in HaCaT cells, while Nav1.5, Nav1.6 and Nav1.7 were highly expressed in fibroblasts. 100 µg/ml *H. hainanum* venom evidently repressed the sodium currents in HaCaT cells. These results indicate that *H. hainanum* venom could affect the function of HaCaT cells partly by inhibiting voltage-gated sodium channels. Moreover, previous studies have also indicated that spider venom can influence the function of immune cells. For example, *Loxosceles* venom induced the activation of blood leukocytes (Manzoni-de Almeida et al., 2018). Our previous studies have demonstrated that *H. hainanum* venom can activate KCa3.1 channels (Huang et al., 2014), which play a key role in the activity of various immune cells (Chiang et al., 2017; Duffy et al., 2015; Matsui et al., 2018). Therefore, we speculated that *H. hainanum* venom may also affect the activities of immune cells.

## Conclusions

In conclusion, these data show that *H. hainanum* envenomation induces edema, inflammation, and hemorrhage. The histological alterations include slight swelling, an intense leukocyte infiltration, and increased microvascular density *in vivo*. Furthermore, venom-induced IL-1β expression and the alteration of cell proliferation, migration, and senescence in HaCaT and fibroblast cells are possible factors that are involved in the pathogenesis of venom-induced inflammatory lesions. These results provide new insights into the mechanisms of the pathology induced by *H. hainanum* venom, contributing to the development of a suitable therapy.

## Additional Information and Declarations

### Data Availability

The following information was supplied regarding data availability: Raw data is available at Figshare: Zhang (2019): 2019.7.16.zip. figshare. Figure. 10.6084/m9.figshare.8872733.v1.
